# Translation, cross-cultural adaptation and validation of the Computer Vision Syndrome Questionnaire into Persian (CVS-Q FA^©^)

**DOI:** 10.1007/s10792-022-02340-3

**Published:** 2022-05-11

**Authors:** Milad Qolami, Ali Mirzajani, Elena Ronda-Pérez, Natalia Cantó-Sancho, Mar Seguí-Crespo

**Affiliations:** 1 Department of Optometry, School of Rehabilitation, Iran University of Medical Sciences, Tehran, Iran; 2 Public Health Research Group, University of Alicante, San Vicente del Raspeig, Alicante, Spain; 3 Biomedical Research Networking Center for Epidemiology and Public Health (CIBERESP), Madrid, Spain; 4 Department of Optics, Pharmacology and Anatomy, University of Alicante, San Vicente del Raspeig, Alicante, Spain

**Keywords:** Computer vision syndrome, Questionnaire, Cross-cultural adaptation, Validation, Occupational health

## Abstract

**Purpose:**

To translate, cross-culturally adapt and validate the Computer Vision Syndrome Questionnaire (CVS-Q^©^) into Persian.

**Methods:**

This study was carried out in 2 phases: (1) the CVS-Q^©^ was translated and cross-culturally adapted into Persian and (2) the validity and reliability of CVS-Q FA^©^ were assessed in a cross-sectional validation study. An expert committee composed of 15 optometrists evaluated content validity (item-level (I-CVI) and scale-level (S-CVI) content validity index were calculated). A pretest was performed (*n* = 20 participants) to verify the comprehensibility of the questionnaire. A total of 102 computer users completed the final questionnaire. Criterion validity and diagnostic performance of the CVS-Q FA^©^ were assessed by calculating sensitivity, specificity and receiver characteristic operator curve. Cronbach's alpha was calculated for the assessment of internal consistency and 46 participants refilled the questionnaire for the second time and the interclass correlation coefficient (ICC) and Cohen's kappa (κ) were evaluated for test–retest reliability.

**Results:**

The translation and cross-cultural adaptation process was performed successfully according to accepted scientific recommendations without any major difficulties. The I-CVI was above 0.80 for all items (symptoms) except item 15 (feeling that sight is worsening) and the S-CVI was 0.92. The CVS-Q FA^©^ showed good sensitivity (81.1%) and acceptable specificity (69.2%). Also, it achieved good internal consistency (Cronbach's alpha = 0.80) and test–retest reliability (ICC = 0.81 and κ = 0.65).

**Conclusion:**

The CVS-Q FA^©^ was successfully translated, cross-culturally adapted, and validated into Persian. This study provides a valid and reliable tool for the assessment of computer vision syndrome among the Iranian working population.

**Supplementary Information:**

The online version contains supplementary material available at 10.1007/s10792-022-02340-3.

## Introduction

In recent decades, the use of digital devices at the workplace has increased significantly. Workers of many occupations usually spend a large proportion of a working day viewing digital screens and moreover with the increase in teleworking due to the COVID-19 disease. Visual demands for working with video display terminals (VDTs) are usually higher than working with conventional hard copy material, and many VDT workers might experience a number of eye- and vision-related symptoms, collectively called computer vision syndrome (CVS) [1] or digital eye strain (DES) [2]. These symptoms include eyestrain, headache, dry eye, blurred vision, diplopia, burning, itching, photophobia, among others [3].

It is estimated that CVS is a very common problem and affects millions of individuals worldwide [2]. However, determining the exact prevalence of this syndrome is challenging. A wide range has been reported among VDT users by different authors from 46.3% to 89.9% [4–8]. The main reason for this considerable difference is variation in criteria and methodologies used to diagnose sufferers [2]. When reviewing the scientific literature, it is observed that most of the published studies use non-validated ad hoc questionnaires [2, 9]. In 2015, Seguí et al. [10] developed and validated the Computer Vision Syndrome Questionnaire (CVS-Q^©^) to evaluate visual and ocular symptoms related to VDT use at work in Spanish population. This questionnaire with good psychometric properties is an acceptable tool to be used both in clinical practice and clinical research settings.

In recent years, the growth of Internet use in Iran has been substantial as a developing county and it is estimated that it has a large number of computer and other digital device users. According to a recent report by the Statistical Center of Iran, 36.8 million (45.5%) of the total population were computer users and 47.9 million (59.1%) were Internet users in 2017 and these figures had increased by 22% and 48.1%, respectively, compared to 2010 [11].

Given the lack of a validated questionnaire in Persian and in order to use CVS-Q^©^ among the Iranian working population, the objective of this study was to carry out the process of translation, cross-cultural adaptation and validation of CVS-Q^©^ into Persian.

## Methods

**Fig. 1:**
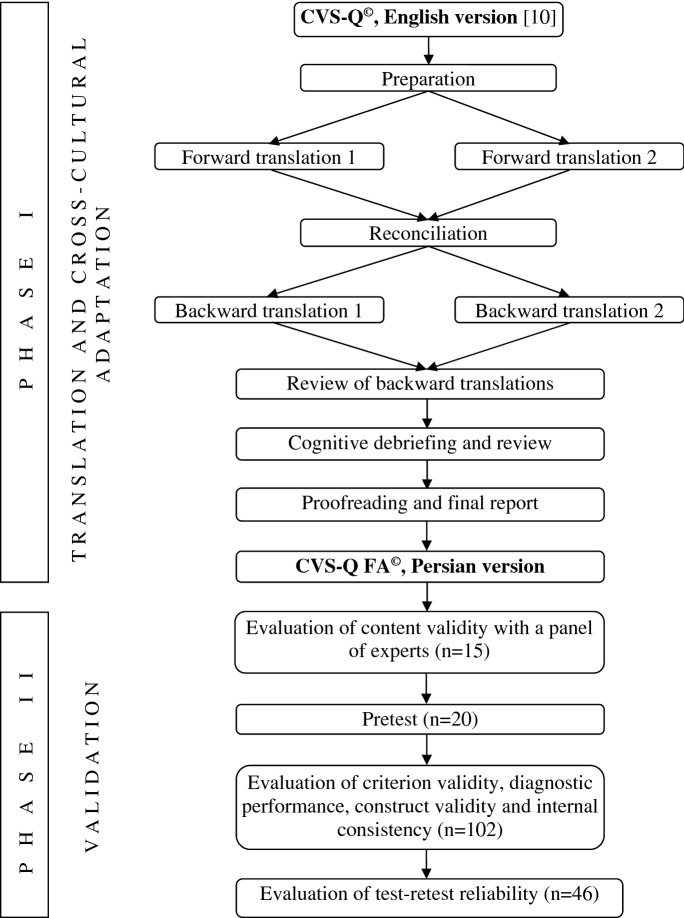
Flowchart of the process of translation, cross-cultural adaptation and validation of CVS-Q^©^ into Persian

This study was carried out in 2 phases. In phase 1, the CVS-Q^©^ was translated and cross-culturally adapted into Persian, and in phase 2, the validity and reliability of the translated questionnaire were assessed in a cross-sectional validation study (Fig. 1).

The CVS-Q^©^ is a simple questionnaire whose design was based on a review of the scientific literature. It was developed with a wide consensus and acceptance among experts from different fields (occupational health, preventive medicine and public health, optometry and ophthalmology), and it was validated through the performance of a pre-test, a pilot test and a reassessment. Likewise, the questionnaire obtained sensitivity and specificity values higher than 70%, good test–retest repeatability, as well as acceptable psychometric properties derived from the Rasch analysis. It is a self-administered questionnaire that evaluates the frequency (never, occasionally and often or always) and intensity (moderate or intense) of 16 ocular and visual symptoms related to VDT use. The frequency and intensity data are recoded to calculate the severity of each symptom, which results in a total score. Total scores ≥ 6 indicate that the subject suffers CVS [10].

### Translation and cross-cultural adaptation process

For phase 1 of this study, we followed the guidelines developed by the Translation and Cultural Adaptation group (TCA group) formed by the International Society for Pharmacoeconomics and Outcomes Research (ISPOR) to create standard guidelines for the translation and cross-cultural adaptation of patient-reported outcome measures (PROMs) [12].

#### Preparation

The permission from the developers of the original questionnaire was obtained, and they agree to participate in the whole process of this study (MMS, ER). A preliminary protocol was shared with both of them, and after a thorough discussion on design and methods, we reached an agreement and the final protocol was obtained.

#### Forward translation

Two independent translations of the English version of CVS-Q^©^ into Persian were performed by two native Iranian bilingual translators (Persian-English), one familiar with the field of medical sciences and the other without any prior knowledge of the field.

#### Reconciliation

Two translated versions in the previous step were compared by Iranian members of the research team (all three fluent in English) and with the help of a third bilingual translator. All discrepancies were discussed, and a reconciled Persian version was generated.

#### Back translation and review

The reconciled Persian version was independently translated back into English by two bilingual translators who were unaware of the study objectives. The translators neither had prior references to the original questionnaire nor prior knowledge of the subject. This step provided a quality control measure for our forward translation step. These two back translations were compared with the original version of the questionnaire by a panel of experts composed of study investigators (both Spanish and Iranian sides, experts in visual and public health) to examine whether the same meaning can be derived from the reconciled version and the original questionnaire. After discussing discrepancies in the translation of some parts of the questionnaire, revisions were applied to the Persian translation and the next reconciled version was obtained.

#### Harmonization

In this step, according to our guidelines, all new translations into other languages are compared with each other and with the original version to ensure that all versions have the same conceptual equivalence. However, because the versions of the CVS-Q^©^ in other languages have been translated from the original version (in Spanish), this step was not necessary.

#### Cognitive debriefing and review

To examine the comprehensibility of the translated version, seven native Iranian VDT workers filled the questionnaire and were interviewed. They were selected so that they adequately represented different sexes, ages and educational levels. During the interview, they were asked about the comprehensibility of different parts of the questionnaire and some alternative terms that might be more appropriate. Then, the review of cognitive debriefing was discussed among study investigators and some minor changes were applied to the questionnaire and the final translation of CVS-Q^©^ in Persian, CVS-Q FA© (پرسشنامه سندرم بینایی کامپیوتر), was obtained (Appendix 1).

#### Proofreading and final report

The CVS-Q FA^©^ was checked carefully by one member of the research team and also a proofreader to find any missing error including type, grammatical and other errors. Eventually, a final report which included the methods of translation and details of decision-making about all parts and items of the questionnaire was produced.

### Validation process

#### Content validity

To assess the content validity of the questionnaire, 15 bilingual optometrists (Persian as the first and English as the second language) were invited to compose a committee of experts. The members of this committee were chosen by snowball sampling and based on their experience related to the subject. A written assessment form was sent via email to the members of the panel. Their qualitative viewpoint was obtained on the instructions and items of the questionnaire. For quantitative evaluation, content validators assessed the clarity and relevancy of all 16 items [13]. Each aspect was assessed by two separate four-point Likert scales, one for relevancy (1 = not relevant, 2 = somewhat relevant, 3 = quite relevant, 4 = highly relevant) and the other for clarity (1 = not clear, 2 = item need some revision, 3 = clear but need minor revision, 4 = very clear), and then, item-level content validity index (I-CVI) was calculated for each item. To do this, the number of experts who rated the item with a 3 or 4 was divided by the total number of experts in the panel. Also, scale level content validity (S-CVI) was calculated by averaging the values of I-CVIs obtained for relevancy. Items with an I-CVI ≥ 0.78 are considered acceptable while those with an I-CVI < 0.78 need revision [14]. The acceptable value for S-CVI is considered 0.90 [14].

#### Pretest

Twenty VDT workers of both sexes and different ages participated in the pretest stage to verify whether they could understand the questionnaire with ease and in a reasonable amount of time. Then, the items which at least 15% of participants experienced difficulties were considered for revision [15].

#### Criterion validity and diagnostic performance of CVS-Q FA^©^

To evaluate the criterion validity of CVS-Q FA^©^ ideally, a gold standard is needed which the instrument can be compared with. But despite the fact that CVS is a widely acknowledged syndrome by clinicians and researchers, there is no gold standard for measuring it. So we used the same criterion that was used by the original developers of the questionnaire: "occurrence of at least one symptom two or three times a week"; this criterion was a reference definition obtained from a literature review [10]. Based on this definition, we categorized participants into sufferers and non-sufferers of CVS.

To determine the diagnostic performance of CVS-Q FA^©^ sensitivity, specificity of all possible values of questionnaire scores was calculated and receiver operator characteristic (ROC) curve was plotted to find a cut-off point that would optimize both sensitivity and specificity.

#### Construct validity of CVS-Q FA^©^

The aim is to determine whether the set of items that constitute the CVS-Q FA^*©*^ has a unidimensional or a multidimensional structure. Mardia's test was performed to assess skewness and kurtosis on the assumption of multivariate normal distribution as it is a test with good power for medium sizes [16, 17]. Bartlett's test of sphericity and the Kaiser–Meyer–Olkin (KMO) test were applied to check for the possible existence of underlying factors and the need for factor analysis. The optimal implementation of the parallel analysis was conducted to determine the number of factors to retain [18], and the polychoric matrix was used instead of the variance–covariance matrix because the CVS-Q FA^*©*^ is a Likert-type scale with fewer than five response options [19]. A Principal Component Analysis was carried out to determine the adequacy of the items to the model and whether any of them should be removed based on the Measure of Sampling Adequacy (MSA) index. Values of MSA < 0.50 suggest that the item does not measure the same domain as the remaining items in the pool, and so that it should be removed [20]. Subsequently, an exploratory factor analysis using the Robust Unweighted Least Squares (RULS) method for factor extraction was carried out because of its higher power at medium sample sizes [21] and allows to carry out the Minimum Rank Factor Analysis (MRFA), which can discriminate the explained common variance from the total common variance and evaluate it separately [18]. The following robust goodness of fit statistics were included to assess the fit of the model: (1) Root Mean Square Error of Approximation (RMSEA), considering as admissible adjustment values ≤ 0.08, (2) Non-Normed Fit Index (NNFI), values > 0.85 would be adequate, (3) Comparative Fit Index (CFI), values > 0.95 would be adequate, (4) Goodness of Fit Index (GFI), values > 0.95 are indicators of a good model fit, (5) Root Mean Square of Residuals (RMSR), using Kelley’s criterion to estimate the reference value to considerer an acceptable adjustment [22], (6) Weighted Root Mean Square Residual (WRMR), values < 1.0 have been recommended to represent good fit [23–27]. In addition, the following indices were also considered at the global level to determine dimensionality: (7) Explained Common Variance (ECV) and (8) Mean of Item residual absolute Loadings (MIREAL). A value of ECV > 0.85 and MIREAL < 0.300 suggests that data can be treated as essentially unidimensional [26].

#### Internal consistency

To evaluate internal consistency, Cronbach’s alpha was calculated. A Cronbach's alpha greater than 0.70 is considered to reflect good internal consistency [28].

#### Test–retest reliability

The two-way mixed effect, single-measure interclass correlation coefficient (ICC) was used to evaluate the test–retest reliability of the scores. ICC was interpreted as following classification based on 95% confidence: < 0.50 poor, 0.50–0.75 moderate, 0.75–0.90 good and > 0.90 excellent [29]. Cohen's kappa (*κ*) was used for the assessment of agreement between the diagnoses. Kappa greater than 0.60 indicates acceptable agreement between measurements [30]. Also, the differences between the means of the test–retest scores obtained were evaluated with Student’s t-test for paired data, and the standard error of measurement (SEM) was calculated with the following Eq. (1):

1 $${\text{SEM}} = \frac{{{\text{SD}} \left( {{\text{standard}}\,{\text{deviation}}} \right)\,{\text{of}}\,{\text{the}}\,{\text{difference}}\,{\text{in}}\,{\text{scores}}}}{\sqrt 2 }$$

#### Procedure and ethical aspects

A convenient sampling method was used at Iran University of Medical Sciences in Tehran, Iran. All university employees and graduate students with at least one hour of work with VDTs per day in the month preceding the study in the workplace were considered for sampling. Considering that between 4 and 10 subjects per item must be taken for assessing questionnaire internal consistency, and as the questionnaire has 16 items, a sample size of 100 is sufficient [31, 32].

This study was approved by the review board and ethics committee of Iran University of Medical Sciences (IR.IUMS.REC.1399.259) and was conducted following the international ethical principles applicable to human research according to the latest revision of the Declaration of Helsinki, and informed consent was obtained from all the participants before taking part in the study.

The exclusion criteria were: (1) Any significant uncorrected refractive error which may be a cause of asthenopia (myopia ≤ -0.75D and manifest hyperopia ≥ 1D, or astigmatism > 0.50D) [1, 33], measured by a Huvitz 8000A auto-refractometer and (2) Any condition and eye disease which may interfere with the symptoms of CVS (trauma, eye diseases, strabismus, amblyopia, surgical intervention and treatments including refractive surgeries).

A total of 130 individuals from the target population gave consent to participate in the study. After taking an ocular history and a preliminary eye examination, by an optometrist, 28 participants were excluded. Sociodemographic (sex, age, educational level) and VDT exposure (hours of use of VDT to work) information was collected from the 102 included computer workers, and the CVS-Q FA^©^ questionnaire was administered; 46 of them agreed to complete the questionnaire for the second time for evaluation of test–retest reliability. The time interval between the two administrations of the questionnaire was 2–4 weeks.

A descriptive analysis of all study variables was performed. Absolute frequency and percentage were calculated for categorical variables. For continuous variables, the mean and SD were obtained. In addition, the total prevalence of CVS was calculated as well as the frequency of the 16 symptoms included in the CVS-Q FA^©^. Floor and ceiling effects were also calculated.

All statistical analyses were performed using SPSS version 25 and FACTOR 10.08.4.

## Results

### Translation and cross-cultural adaptation

Two forward translations were quite similar to each other and the reconciled version was obtained by complete agreement upon most parts of instruction and items, although some doubts on few parts of the questionnaire remained to be resolved in the next steps of the process. After carrying out the backward translation step, the discrepancies between the original questionnaire and translated versions were discussed. At this point, the original developers emphasized that few parts of the translation should be changed to be consistent with the original version of CVS-Q^©^. Also, other differences were discussed and the final decisions were made (Table 1).

**Table 1:** Description of major discrepancies between the translated version and original questionnaire and their solutions

Issue	Discrepancies	Solution
"Indicate whether you experience any of the following symptoms during the time you use the computer at work. For each symptom, mark with an X"	In backward translation, the word "symptoms" in this part of instruction, was translated to "problems" which did not represent the intention of the original questionnaire	Persian translation was altered so that when it was back-translated it meant "symptoms"
"Difficulty focusing for near vision"	When the item was back-translated into English, it was changed to "blurred vision problem at near distance" which was not an appropriate equivalent of the original item	Persian translation was changed to a sentence that meant "difficulty in sharpening the image at near distance" which was a better equivalent of the original item
"Feeling that sight is worsening"	In the translation process, "worsening" was interpreted as "deceased" which was not the intention of the original questionnaire	The item was retranslated so that in the back-translation, it was changed to "worsening"

The review of cognitive debriefing revealed that all 7 computer users had a good overall comprehension of the questionnaire. However, some observations about completed questionnaires and comments from participants led to 2 minor adjustments in the instruction of the questionnaire. First, 3 of the participants recorded their symptoms not just during the computer use but also other times of the day so it was decided to underline "during the computer use" in the instruction. Second, 2 out of seven users forgot to mark the intensity during self-completion of the questionnaire; therefore, to emphasize that two features must be marked for each symptom, the words "frequency" and "intensity" were underlined in the instructions.

### Validation

#### Content validity

From a qualitative point of view, the expert committee evaluated the overall comprehensiveness of the instrument as acceptable, and therefore, no major changes were needed. On the other hand, for quantitative evaluation of content validity, 15 members of the expert committee rated the items of the questionnaire for clarity and relevancy. The S-CVI of the overall instrument was 0.92 and 15 items had an I-CVI ≥ 0.80 and the only item that had an I-CVI lower than acceptable was item 15 (feeling that sight is worsening). After discussing the suggestions from the expert committee, item 15 was modified by replacing the sentence meant "feeling that your vision has been worsening" with" feeling that your vision is worsening." After a reassessment of item 15, all items of the instrument reached the I-CVI threshold ≥ 0.78. Table 2 shows the final I-CVI of 16 items of CVS-Q FA^©^.

**Table 2:** Item-level content validity index for 16 items of the Computer Vision Syndrome Questionnaire, Persian version

Item	Symptom	I-CVI for relevancy	I-CVI for clarity
1	Burning	1	1
2	Itching	0.93	1
3	Feeling of a foreign body	0.86	0.93
4	Tearing	0.80	1
5	Excessive blinking	0.80	1
6	Eye redness	1	1
7	Eye pain	1	0.93
8	Heavy eyelids	0.80	0.86
9	Dryness	1	1
10	Blurred vision	1	1
11	Double vision	0.80	1
12	Difficulty focusing for near vision	0.86	0.86
13	Increased sensitivity to light	0.86	0.86
14	Colored halos around objects	0.86	0.93
15	Feeling that sight is worsening	0.93	0.93
16	Headache	1	1

*I-CVI* Item level content validity index

#### Pretest

The CVS-Q FA^©^ with a similar graphical design as the original version was applied to 20 computer users in the workplace (12 females and 8 males) with a mean age of 38.5 ± 10.0 years. The mean time for the completion of the questionnaire was 3.1 ± 1.7 min. All participants showed little difficulties in understanding and self-completion of the questionnaire.

#### General characteristics of the study population who has been involved in the assessment of criterion and diagnostic performance of CVS-Q FA^©^, construct validity and internal consistency

The total number of subjects included in the study were 102 computer users with a mean age of 40.0 ± 11.0 years (mean ± SD), including 63.7% females. The average exposure to VDT was 5.0 ± 2.2 h per day, with a range between 1 and 10 h. The description of the sample is shown in Table 3.

**Table 3:** Distribution of the sample (*n* = 102) according to sociodemographic and exposure to digital devices characteristics

Variable	*N* (%)
Total	102 (100)
*Age (years)*	
20–29	27 (26.5)
30–39	29 (28.1)
40–49	25 (24.5)
50–59	18 (17.7)
≥ 60	3 (2.9)
*Sex*	
Female	65 (63.7)
Male	37 (36.3)
*Education level*	
High school graduate	12 (11.8)
Bachelor	34 (33.3)
Master	35 (34.3)
Doctorate	21 (20.6)
*Use of work VDT (hours/day)*	
< 2	6 (5.9)
2–3.5	26 (25.5)
4–5.5	30 (29.4)
6–7.5	18 (17.6)
8–10	22 (21.6)

*VDT* video display terminal

#### Criterion validity and diagnostic performance of CVS-Q FA^©^

**Fig. 2:**
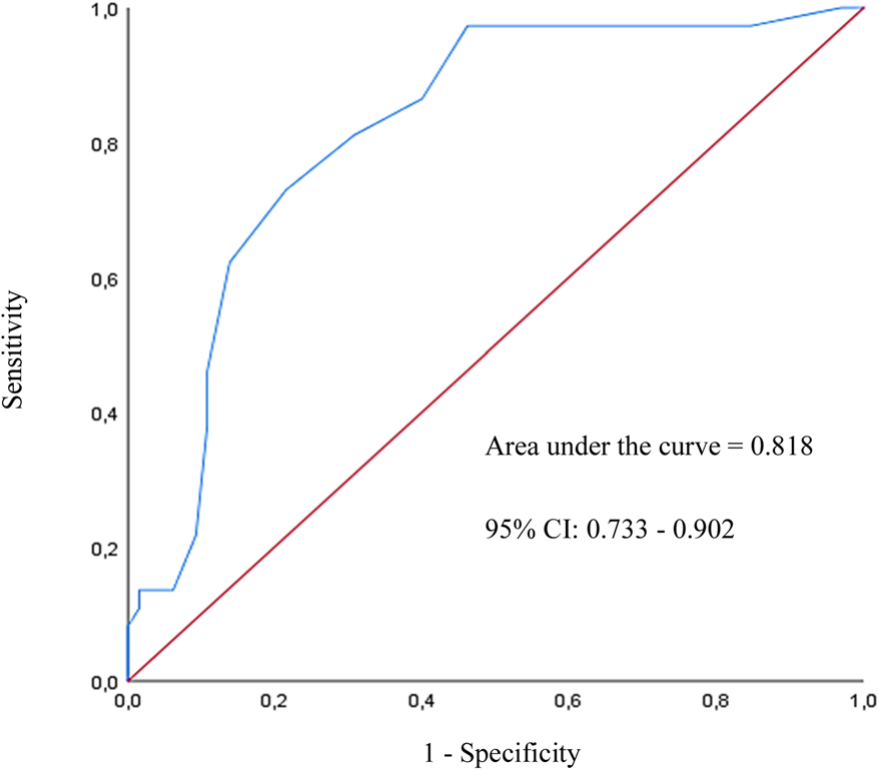
Receiver operator charactristic curve of the Computer Vision Syndrome Questionnaire, Persian version

According to the plotted ROC curve (Fig. 2), a cutoff point of 7 would optimize both sensitivity and specificity with values of 81.1% and 69.2%, respectively. So, computer users who obtain 7 points or more on completing the questionnaire would be considered as CVS sufferers. According to this cutoff value, 50 individuals (49%) of the study population had CVS. Also, the area under the ROC curve (AUC = 0.818; 95% CI of 0.733, 0.902 and a *p* < 0.001) indicates that CVS-Q FA^©^ has an acceptable diagnostic performance.

#### Construct validity of CVS-Q FA^©^

The results of the Mardia’s test indicate that the distribution of the results is not normal; skewness (66.95, *p* = 1.000); and kurtosis (299.14, *p* < 0.01). The Bartlett's statistic result was *p* = 0.00001 (*p* < 0.05), which indicates that there is a relationship between the items, and in the KMO test a value of 0.78 was obtained, which indicates that the relationship between the items is regular. Therefore, the two assumptions for applying factor analysis are met.

The parallel analysis resulted in one factor, and the exploratory factor analysis also extracted a single factor accounting for 39.84% of the explained common variance. The MSA values ranged from 0.67 (item 12) to 0.85 (item 13), so no items were dropped from the model. The appropriateness was verified by the robust goodness of fit statistics, which obtained the following results: RMSEA = 0.075, NNFI = 0.957, CFI = 0.963, GFI = 0.925, RMSR = 0.125, WRMR = 0.089. The ECV statistic = 0.824 and the MIREAL statistic = 0.218 were obtained. All factor loadings were > 0.30, ranging from 0.33 (item 4) to 0.86 (item 15).

#### Internal consistency and Test–retest reliability

Floor and ceiling effect analyses were performed, and neither were observed as 2% and 0% of participants obtained the lowest (0) and highest (32) possible score of the questionnaire, respectively.

The CVS-Q FA^©^ showed acceptable internal consistency (Cronbach's alpha was 0.80), and no item deletion could change the internal consistency significantly (as α ranged between 0.78 and 0.82 after deletion of each item). ICC and κ were used to assess the test–retest reliability and they were equal to 0.81 and 0.65, respectively, which are considered as good and acceptable. Likewise, a SEM = 1.915; 95% CI of 3.06, 10.56 was obtained.

### Ocular and visual symptomatology evaluated with the CVS-Q FA^©^

**Fig. 3:**
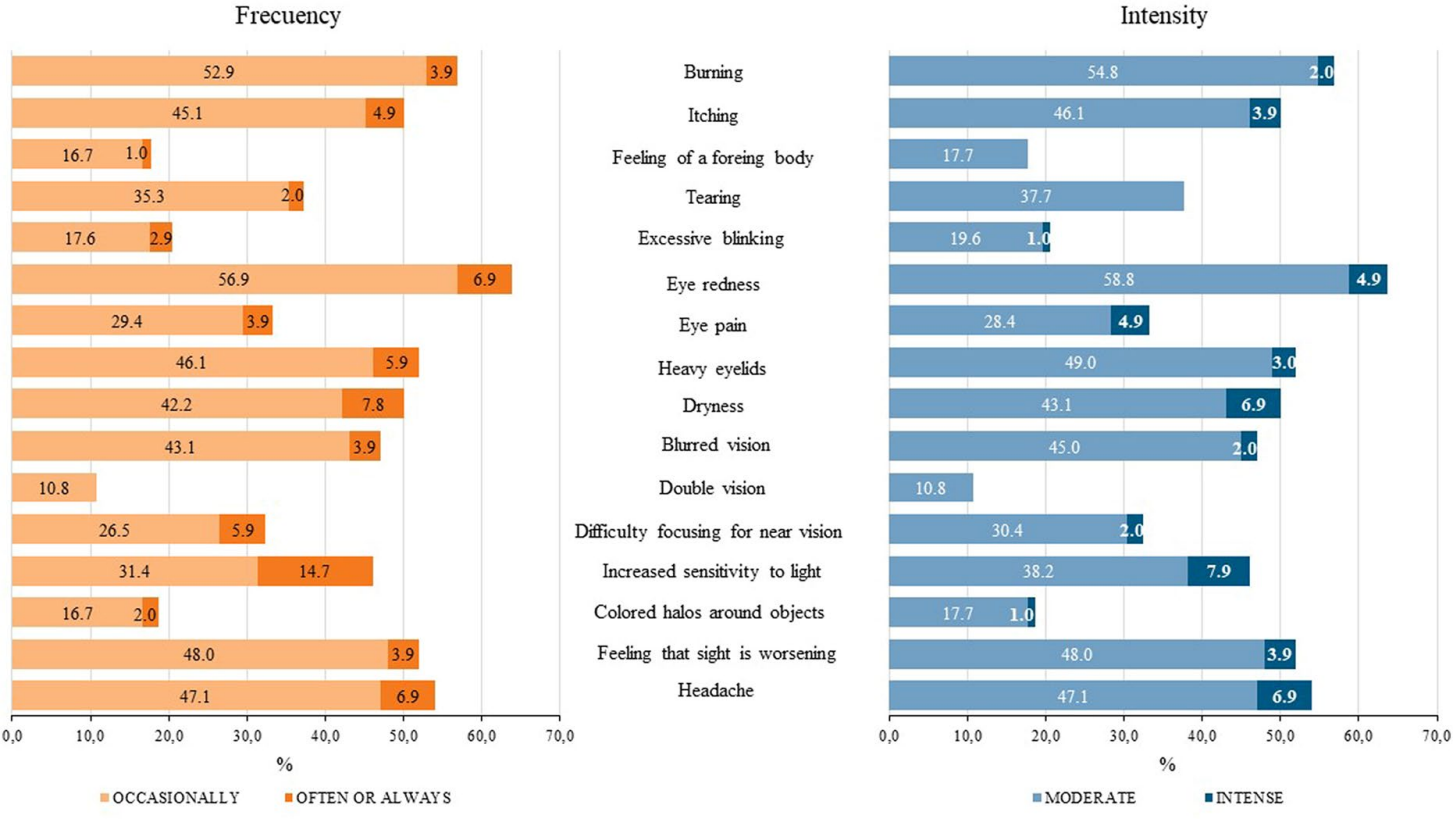
Prevalence of symptoms of the Computer Vision Syndrome Questionnaire, Persian version, according to frequency and intensity

Figure 3 shows that "eye redness," "burning" and “headache” were the most frequent symptoms (63.8%, 56.8% and 54.0%, respectively) and "double vision," "feeling of a foreign body" and “colored halos around objects” were the least frequent symptoms (10.8%, 17.7% and 18.7%, respectively). Almost all symptoms occurred occasionally; however, the symptom "increased sensitivity to light" was often or always present almost in 15% of the sample analyzed. In general, the intensity of symptoms was moderate.

## Discussion

In this study, we have carried out the translation, cross-culturally adaptation and assessed the measurement properties of the Persian version of the CVS-Q^©^. The overall results were good with respect to cognitive debriefing, comprehensibility, face, content, criterion and construct validity, internal consistency and reliability.

To date, the original CVS-Q^©^ has been translated and cross-culturally adapted to different languages: English, Slovak and Italian [10, 34, 35], following the methodology developed by Beaton et al. [36] which is also recommended by other authors [15]. This represents a methodological difference with respect to the ISPOR standard guidelines followed in this study for the Persian version [12]. Although one of the most widely used methods for PROMs was the guidelines recommended by Guillemin et al. in 1993 [37], which were updated and presented more formally in 2000 [36], the ISPOR standards guidelines is a newly developed and detailed process [12]. In a recent review of the literature, 31 different cross-cultural adaptation methods were identified and no consensus was found. The authors of this review indicated that most of guidelines would achieve comparable results, and choosing one is a matter of preference and logistic, but there is lack evidence of the superiority of one method over another [38]. In our study, the process of translation and cross-cultural adaptation was performed without major difficulties. Partially, this may be due to the simplicity of the original questionnaire and to the fact that we rigorously followed the steps established in the literature [12].

Nevertheless, there is a lack of consensus on how to carry out the back translation step. Several authors indicate that involving only professional translators in this step has no clear scientific basis [39, 40]. It is proposed as an alternate approach to carry out a dual panel consisting of a team of professional translators working under an experienced bilingual coordinator and a monolingual focus group in the target language [41]. However, a comparative study concluded that although the target population prefers dual panel approach, there were no obvious differences in the psychometric performance of the questionnaires obtained [42]. Thus, both methodologies are able to produce a reliable adapted instrument [38]. In our study, we decided to carry out the back translation step following the conventional methodology (two professional translators) since it is a widely implemented stage [12, 36], and which remains fundamental when the committee has inadequate proficiency in the source and target language [43]. In fact, it was also decided to incorporate Spanish and Iranian experts in visual and public health in the review after the back translation step. Since the guidelines recommended by Beaton et al. [36] include a multidisciplinary expert committee review, an experimental study concludes that translations reviewed by multidisciplinary committee improve the face and content validity of the adapted tool [43].

Results of cognitive debriefing showed that VDT users could understand the questionnaire easily, and the pretest confirmed that they could complete it within a reasonable amount of time, with a similar average completion time that the original version of the questionnaire [10]. Only a few modifications were applied to the instruction of the questionnaire to prevent any misunderstanding about the time of occurrence and rating scales of the symptoms.

There are different initiatives that propose quality standards to assess the measurement properties of instruments that evaluate health status, such as the Scientific Advisory Committee of the Medical Outcomes Trust (SAC-MOS) [44], the criteria proposed by Terwee et al. [32], the Consensus-based Standards for the selection of health Measurement INstruments (COSMIN) [45] and those by Bopaire et al. [28]. The terminology and definitions used in these initiatives differ slightly. In fact, COSMIN [45], SAC-MOS [44] and Bopaire et al. [28] consider internal consistency as a subcategory of reliability, whereas for Terwee et al. [32] they are different properties, and SAC-MOS also includes methods based on item response theory (IRT) [44]. In this study, it is followed the process of psychometric evaluation of a questionnaire recommended by Bopaire et al. [28].

Our results of the cognitive debriefing did not identify any significant changes indicating that the CVS-Q FA^©^ measures what it is intended to measure in the judgement of the experts and the target population, which is essential before assessing the other properties of the questionnaire [46]. During the process of content validity assessment and after modifying item 15 (feeling that sight is worsening), all the 16 items of the questionnaire met the criterion of I-CVI ≥ 0.78 [14]. This demonstrated good agreement between content validators on the clarity of translation and the necessity of all the items in the instrument. In this study, we also evaluated the criterion validity and diagnostic performance of CVS-Q FA^©^ by determining the sensitivity, specificity, as well as the area under the ROC curve. The CVS-Q FA^©^ obtained sensitivity and specificity like those obtained by authors of the original questionnaire (sensitivity = 75%; specificity = 70.2%; AUC = 0.826, with a 95% CI of 0.779, 0.874 and a *p* < 0.001) [10]; however, the cutoff point of this version is higher than the original questionnaire. Nevertheless, slight changes in the cut-off point are common in different linguistic versions of health questionnaires [47, 48]. Factor analysis confirmed the one-dimensional structure of the scale, no need to remove any items from the scale and good model fit due to the robust goodness of fit statistics, same result as the original scale but in this case by factor analysis rather than Rasch analysis. The evaluation of the internal consistency was acceptable, and test–retest analysis revealed also good stability over time of the questionnaire, both similar to the results of the original CVS-Q^©^ [10].

During the process of validation, CVS-Q FA^©^ was applied to a sample of university staff and graduate students who regularly used computers in their workplace. The results showed that CVS is a common problem among this population as 49% of participants were diagnosed as CVS sufferers (findings already published) [49]. This observation agrees with previous studies that have reported a high prevalence of CVS, ranged between 54.6% and 73%, among computer users in the workplace [6, 7, 50]. In our study, the most prevalent symptoms were eye redness (63.8%), burning (56.8%) and headache (54.0%). These three symptoms are relatively frequent among digital devices users [1, 3, 51, 52]. The participants of this study were typical VDT users in the office and academic environment in Iran; therefore, this relatively high prevalence of CVS and symptoms was expected. This is consistent with hypotheses based on the assumption that the instrument validly measures the construct to be measured. Floor and ceiling effects are demonstrated when > 15% of participants obtain lowest of highest score on completing the questionnaire [32], so in our study these effects are not observed.

There are some limitations to this study that should be noted. For translation and cross-cultural adaptation, backward translations should have been carried out by English natives who were also fluent in Persian. Unfortunately, we could not find such persons with these characteristics, so this step was carried out by bilingual Persian translators who had spent some years in an English-speaking country. Moreover, as is the case in other specialties where there is still no gold standard [53], the difficulty in assessing the criterion validity of an instrument is greater. In our case, and trying to be as conservative as possible, we have followed the same criteria used by the original developers of the questionnaire [10]. In addition, it would be desirable to have a larger sample size to carry out the factor analysis, but this limitation has been addressed by using robust analysis techniques. Finally, it should be noted that subjective refraction and accommodative and binocular tests were not carried out on the participants in this study. Symptoms derived from small uncorrected refractive errors or untreated accommodative and binocular dysfunctions could be confounding some symptoms of CVS. Therefore, the prevalence data reported in the Iranian population should be taken with caution. The main strength of the present study is that all steps of the process of translation, cross-cultural adaptation and validation of CVS-Q FA^©^ were performed in collaboration with developers of the original version of the questionnaire to make sure that the translated version is optimally consistent with the original version. Also, to assess the reliability we carried out a preliminary eye examination to exclude those participants with a significant uncorrected refractive errors and eye diseases trying to reduce interfering symptoms as much as possible, although some confounding factors were inevitable. It has been attempted that the symptoms reported by workers were due to computer use, not other factors.

## Conclusions

In conclusion, the original CVS-Q^©^ questionnaire was successfully translated, cross-culturally adapted and validated into Persian. The findings of this study show that CVS-Q FA^©^ is a questionnaire with acceptable psychometric properties, easy to understand by computer users and can be completed in a short amount of time. In future studies and given the large number of digital devices users in Iran, this questionnaire could be used for investigating different aspects of CVS among general population, in groups of computer users with individual or job characteristics that increase the risk of CVS, as well as in other digital device users, such as mobile users or video game players. Aspects such as prevalence, economic consequences, causes and preventive measures should be analyzed in relation to CVS in the Iranian population. Especially now that it seems that the prevalence of CVS is growing because of the COVID-19 pandemic due to the increase in use of electronic digital devices [54].

## Supplementary Information

Below is the link to the electronic supplementary material.

Supplementary file1 (PDF 225 KB)

## Abbreviations

VDT: Video display terminal
I-CVI: Item-level content validity index
