# Supplementary material for: Translation, cross-cultural adaptation and validation of the Computer Vision Syndrome Questionnaire into Persian (CVS-Q FA©)
Source: Int Ophthalmol. 2022 May 11;42(11):3407–20. doi: 10.1007/s10792-022-02340-3 (PMC9092937; doi:10.1007/s10792-022-02340-3)
Supplement: Supplementary file 1 — Supplementary file1 (PDF 225 KB) [file 10792_2022_2340_MOESM1_ESM.pdf]

پرسشنامه سندرم بینایی کامپیوتر

CVS-Q© FA
(COMPUTER VISION SYNDROME QUESTIONNAIRE)

توسط کاربر تکمیل شود.

مشخص کنید کدامیک از علایم زیر را در هنگام استفاده از کامپیوتر در محل کار خود دارید. برای هر علامت دو مشخصه را در جدول زیر علامت بزنید.

الف) تناوب، یعنی چندبار اتفاق می افتد، به این معنا که :

هیچ وقت = این علامت هیچ گاه اتفاق نمی افتد

بعضی اوقات = به صورت گهگاهی یا پراکنده یا یک بار در هفته

اغلب یا همیشه = دو یا سه بار هفته یا تقریبا هر روز

ب) شدت.

توجه داشته باشید که اگر تناوب هر یک از علایم را هیچ وقت انتخاب کرده باشید نباید شدت را علامت بزنید.

	تناوب			شدت	
	هیچ وقت	بعضی اوقات	اغلب یا همیشه	متوسط	شدید
۱- سوزش چشم					
۲- خارش چشم					
۳- احساس جسمی خارجی در چشم					
۴- اشک ریزش					
۵- پلک زدن زیاد از حد					
۶- قرمزی چشم					
۷- درد چشم					
۸- احساس سنگینی در پلک ها					
۹- خشکی چشم					
۱۰- تاری دید					
۱۱- دوبینی					
۱۲- مشکل در واضح کردن تصویر در فاصله ی نزدیک					
۱۳- افزایش حساسیت به نور					
۱۴- دیدن هاله های رنگی اطراف اجسام					
۱۵- احساس اینکه دید شما بدتر شده					
۱۶- سردرد					

توسط محقق تکمیل شود.

با در نظر گرفتن ملاحظات زیر نمره ی کل را محاسبه کنید.

- **تناوب**
  - **هیچ وقت = ۰**
  زیرثبت شود:
  - **بعضی اوقات = ۱**
  ۰=۰
  - **اغلب یا همیشه = ۲**

- **شدت :**
  - **متوسط = ۱**
  - **شدید = ۲**

- **حدت :**
  - حاصل تناوب x شدت می بایست به صورت
  ۲=۴ ؛ ۱= ۲ یا ۱ ؛

	تناوب	شدت	تناوب x شدت	حدت
۱- سوزش چشم				
۲- خارش چشم				
۳- احساس جسم خارجی در چشم				
۴- اشک ریزش				
۵- پلک زدن زیاد از حد				
۶- قرمزی چشم				
۷- درد چشم				
۸- احساس سنگینی در پلک ها				
۹- خشکی چشم				
۱۰- تاری دید				
۱۱- دوبینی				
۱۲- مشکل در واضح کردن تصویر در فاصله نزدیک				
۱۳- افزایش حساسیت به نور				
۱۴- دیدن هاله های رنگی اطراف اجسام				
۱۵- احساس اینکه دید شما بدتر شده				
۱۶-سردرد				
			نمره ی کل:	$\sum_{i=1}^{16} =$

اگر نمره کل ≥ ۷ ، تشخیص سندرم بینایی کامپیوتر برای کاربر صورت می گیرد.
